# Biomarkers of fibrosis, kidney tissue injury and inflammation may predict severity and outcome of renal ANCA – associated vasculitis

**DOI:** 10.3389/fimmu.2023.1122972

**Published:** 2023-03-20

**Authors:** Veronika Satrapova, Nadja Sparding, Federica Genovese, Morten Asser Karsdal, Lenka Bartonova, Doubravka Frausova, Eva Honsova, Marek Kollar, Miloslav Suchanek, Helena Koprivova, Romana Rysava, Vladimira Bednarova, Vladimir Tesar, Zdenka Hruskova

**Affiliations:** ^1^Department of Nephrology, First Faculty of Medicine, Charles University and General University Hospital in Prague, Prague, Czechia; ^2^Nordic Bioscience, Herlev, Denmark; ^3^Department of Pathology, Institute for Clinical and Experimental Medicine, Prague, Czechia; ^4^Faculty of Environment, Jan Evangelista Purkyně University in Ústí nad Labem, Ústí nad Labem, Czechia; ^5^Institute of Medical Biochemistry and Laboratory Diagnostics, First Faculty of Medicine, Charles University and General University Hospital in Prague, Prague, Czechia

**Keywords:** ANCA- associated vasculitis, biomarkers, DKK-3, kidney biopsy, kidney fibrosis, chronic kidney disease, PRO-C6

## Abstract

**Background:**

Activity and chronicity of kidney involvement in ANCA-associated vasculitis (AAV) can be currently reliably evaluated only by kidney biopsy. In this study, we measured a panel of serum and urinary biomarkers collected at the time of kidney biopsy and hypothesized that they could reflect specific histopathological parameters in the biopsy and help to predict prognosis.

**Methods:**

We examined a cohort of 45 patients with AAV and 10 healthy controls. Biomarker levels (DKK-3, CD163, EGF, PRO-C6 and C3M) were measured in this study by ELISA. Biopsies were scored with a scoring system for AAV (focal x crescentic x sclerotic x mixed class) and interstitial fibrosis was quantified.

**Results:**

Levels of urinary DKK-3, CD163, EGF, PRO-C6 and C3M significantly differed among biopsy classes in AAV, with urinary DKK-3 and PRO-C6 levels being highest in the sclerotic class and lowest in the focal class, urinary CD163 levels highest in the crescentic class and urinary C3M levels highest in the focal class. Moreover, the urinary biomarkers were able to discriminate focal biopsy class from the other classes. Urinary DKK-3, EGF, PRO-C6 and C3M levels measured at the time of biopsy were also significantly related to the extent of fibrosis and to the final kidney function at the end of follow-up.

**Conclusions:**

This small pilot study suggests that selected urinary biomarkers of fibrosis and inflammation may reflect changes in the kidney biopsy and be prognostic of kidney outcome in patients with AAV.

## 1 Introduction

The anti-neuthrophil cytoplasmic antibody (ANCA) – associated vasculitis (AAV) is a rare multisystemic autoimmune disease characterized by the presence of ANCA and necrotizing inflammation of small vessels (1, 2).

AAV is a severe disease involving mainly respiratory tract and the kidneys, with progression to end-stage kidney disease (ESKD) in 20-30% of cases within 5 years (3, 4).

Kidney biopsy is the gold standard for establishing the diagnosis and for assessing the disease activity/chronicity stage of ANCA associated glomerulonephritis (5). However, the kidney biopsy is invasive, associated with a risk of bleeding and usually requires a short hospital stay. Furthermore, in some patients it may not be feasible because of the severity of the disease, need for intensive care treatment or other comorbidities.

Kidney fibrosis is a typical feature of chronic kidney disease (CKD) present also in patients with AAV. It is caused by an imbalance in turnover of extracellular matrix (ECM) components. ECM biomarkers describing the morphological changes in the kidney tissue are detectable in serum and urine, potentially even in patients with still preserved kidney function (6).

Novel serum and, especially, urinary biomarkers able to assess the type, extent and potentially prognosis of kidney disease in AAV are needed. In this study, we aimed to assess the potential value of a panel of fibrosis, kidney tissue injury and inflammation biomarkers to diagnose and assess kidney disease in AAV.

Several biomarkers of the morphological changes in the kidney tissue have been already described. DKK-3 (Dickkopf-related protein 3), a stress-induced tubular epithelia-derived profibrotic glycoprotein, was shown to be prognostic for risk of short-term eGFR loss regardless of the cause of kidney injury and specifically correlated with the extent of tubular atrophy and interstitial fibrosis (6–8). High levels of urinary soluble CD163 (a specific macrophage activation marker) were significantly associated with active renal vasculitis (9, 10).EGF (epidermal growth factor), a specific tubular marker, when measured in urine, could assess the degree of tubular damage and predict chronic kidney disease progression in patients with AAV (11–13).

PRO-C6 and C3M are two markers of collagen turnover that have been previously shown to strongly correlate with kidney function and associate with adverse kidney- and cardiovascular-related outcomes in patients with CKD of different aetiology (14–17). Increased levels of serum and urine PRO-C6 were previously associated with more advanced degrees of interstitial fibrosis in patients with IgA nephropathy, ANCA vasculitis and lupus nephritis (18, 19). Decreased levels of urinary C3M were associated with a higher extent of fibrosis in lupus nephritis patients (19).

The aim of this study was to investigate the association of DKK-3, CD163, EGF, PRO-C6 and C3M measured in urine with the histological changes in kidney biopsies (including both glomerular and interstitial changes), as well as their prognostic potential for kidney function in patients with AAV. To our knowledge, this is the first time these biomarkers have been studied together in a cohort of AAV patients.

## 2 Methods

### 2.1 Subjects

The cohort consisted of 45 patients with AAV (M/F 27/18, median age 63 (range 28-81) years, type of ANCA PR3 (proteinase - 3)/MPO (myeloperoxidase)/double 20/23/2) sampled on the day of kidney biopsy, and 10 healthy controls (M/F 1/9, median age 41 (range 26-48) years). All patients were recruited from Department of Nephrology of the General University Hospital in Prague, Czech Republic. Follow-up clinical data were available at 1 year in 44 patients and at 3 years in 32 patients. Two mentioned biomarkers (DKK3 and PRO-C6) were measured in the same cohort of patients in our previous research study (18).

All patients received standard immunosuppressive therapy, including corticosteroids and cyclophosphamide, immediately after the diagnosis of AAV, and were later switched to maintenance therapy (mostly with azathioprine). One year after diagnosis, all patients were still treated with maintenance immunosuppressive therapy, and after 3 years the immunosuppressive therapy was completely withdrawn in 6% of the patients.

The study was conducted in accordance with the World Medical Association’s Declaration of Helsinki. The participants provided their written informed consent to participate in this study and the study was reviewed and approved by the Ethics Committee of General University Hospital in Prague.

### 2.2 Data collection

Basic clinical and laboratory data were obtained from routine patients’ visits (including serum creatinine, proteinuria, ANCA levels) at the time of diagnosis and during follow-up (1 and 3 years after diagnosis and the last visit, the median time of follow-up was 38 months). Serum and urine samples were collected and stored in the Biobank at -80°C. According to the median of serum creatinine (156 µmol/l) at the end of follow-up, we divided patients into two groups – the favourable prognosis group with the last available serum creatinine below 156 µmol/l and the unfavourable prognosis group with the last serum creatinine above 156 µmol/l (including dialysis).

### 2.3 Assays

The selected biomarkers were measured in paired serum and urine samples using commercially available enzyme-linked immunosorbent assay (ELISA). DKK-3 was measured in serum and urine with the human ELISA kit by Sigma-Aldrich. CD163 and EGF were measured in urine with ELISA kits by R&D systems. All of them were measured in the laboratories of the General University Hospital in Prague. PRO-C6 and C3M levels were measured in serum and urine with ELISA kits by Nordic Bioscience, Denmark. Serum and urinary samples used for measurement of these biomarkers were not available for all 45 patients. Finally, we had 45 samples in AAV group measured for the basic comparison with healthy controls, 40 samples for measuring biomarkers in particular biopsy classes used in most subanalyses, and 38 samples for measuring renal outcome. Urinary marker levels were normalized to urinary creatinine measured by standard measurement method in the Institute of Medical Biochemistry and Laboratory Diagnostics in the General University Hospital in Prague.

### 2.4 Kidney biopsy

Ultrasound-guided kidney biopsies were performed at the time of diagnosis of AAV at the General University Hospital in Prague.

The glomerular morphology in the biopsies was classified according to Berden’s scoring system for AAV as focal (> 50% of normal glomeruli), crescentic (> 50% of glomeruli with cellular crescents), sclerotic (> 50% of globally sclerotic glomeruli) or mixed (meeting none of the above criteria) (5). The Banff classification was used to quantify interstitial fibrosis. Interstitial fibrosis in the cortical area was classified as: ci0 – in up to 5%, ci1 – in 6-25% (mild), ci2 – in 26-50% (moderate) and ci3 – in > 50% (severe) (20).

### 2.5 Statistical analyses

All biomarkers were measured in both serum and urine samples but only urine biomarkers were kept in the final statistical analyses for their non-invasive character.

For the comparison of various groups, the non-parametric tests were used. Two groups may be compared by Mann-Whitney test, for more than two groups the Kruskal-Wallis test was used.

The p-value is obtained using the asymptotic approximation of the distribution of the statistic. The estimation of p-values is included in software XLSTAT.

Linear discriminant analysis was used for the calculation of ROC only. All results were confirmed by the Logistic regression.

Linear discriminant analysis predicts a membership in a group or category based on observed values of several continuous variables (21). Specifically, discriminant analysis predicts a classification Y variable (e.g. ANCA classes) based on known continuous responses X (biochemical, clinical, histological markers). The data for a discriminant analysis consists of a sample of observations with known group membership together with their values on the continuous variables. We used discriminant analysis with transformed variables, the so-called principal. To verify the correct discriminant ability, confusion matrix was used, which resulted in classifying each of the objects in those categories.

Logistic regression is a frequently used method, as it enables binary variables, the sum of binary variables, polytomous variables (variables with more than two categories), or ordinal variables (polytomous variables with ordered values) to be modeled. The principle of the logistic regression model is to link the occurrence or non-occurrence of an event to explanatory variables. For logistic regression, the dependent variable (y), also called the response variable, follows a Bernoulli distribution for parameter p (p is the mean probability that an event will occur) when the experiment is repeated once. The probability parameter p is here a linear combination of explanatory variables, x_i_. The most common functions used to link probability p to the explanatory variables, x_i_ (i=1,n), are the logistic function (the Logit model). XLSTAT uses the Newton-Raphson algorithm to iteratively find a solution. All statistical analysis were performed by XLSTAT software.

## 3 Results

### 3.1 Baseline characteristics of the study cohort

The demographic, clinical and laboratory data on participants are summarized in **Table 1**. All patients with AAV had biopsy-confirmed kidney involvement, often severe, with 22% of them requiring dialysis at the time of diagnosis.

**Table 1:** Baseline characteristics of study subjects at entry.

	AAV	Healthy
**N**	45	10
**Sex (M/F) N, %**	27/18 (60/40%)	1/9 (10/90%)
**Age (median (range)) years**	63 (28-81)	41 (26 – 48)
**Type of ANCA (PR3/MPO/double) N, %**	20/23/2 (44/51/4%)	
**Serum-creatinine in non-dialysed** **(median (range)) umol/l**	165 (58-614)	
**GFR CKD-EPI in non-dialysed** **(median (range)) ml/s/1.73 m^2^**	0.47 (0.12–1.95)	
**No. of patients on dialysis N, %**	10 (22%)	
**Proteinuria (median (range)) g/day**	1.39 (0.2-8)	
Organ involvement (N, %)		
- **Kidney**	45 (100%)	
- **Lungs**	22 (49%)	
- **ENT**	7 (16%)	
ANCA class (N, %)		
- **Focal**	13 (29%)	
- **Mixed**	9 (20%)	
- **Crescentic**	9 (20%)	
- **Sclerotic**	11 (24%)	
- **NA**	3 (7%)	
Banff score (N, %)		
- **ci0**	5 (11%)	
- **ci1**	22 (49%)	
- **ci2**	7 (16%)	
- **ci3**	7 (16%)	
- **NA**	4 (8%)	

"NA" not available data.

### 3.2 Comparison of selected biomarkers between AAV and healthy controls

Using discrimination analysis, a combination of 5 urinary markers (DKK-3, CD163, EGF, PRO-C6 and C3M) could significantly differentiate patients with AAV from healthy controls with 100% accuracy of discrimination. Urinary DKK-3, CD163 and PRO-C6 levels were higher and urinary EGF and C3M levels were lower in patients with AAV as compared with healthy controls. The medians and I. and III. quartile of biomarkers levels in patients with AAV and healthy controls are summarized in **Table 2**.

**Table 2:** Levels of urinary biomarkers in AAV patients and healthy controls.

Urinary biomarkers	AAV (N = 45)	Healthy (N = 10)	p-values
**DKK-3 (ng/mmol)**	614 (268;1637)	60 (21.2;74.9)	**<0.0001**
**CD163 (ng/mmol)**	1237 (358;2309)	0.0 (0.0;0.0)	**<0.0001**
**EGF (ng/mmol)**	161 (78;335)	895 (607;1097)	**0.001**
**PRO-C6 (ng/mg)**	22.3 (5.9;58.5)	3.1 (2.3;3.6)	**<0.0001**
**C3M (ng/mg)**	41(22;60)	66 (58;81)	**0.007**

Data are presented as median and I. and III. quartile.

The bold p-values are significant.

### 3.3 Association of biomarkers with renal biopsy findings

**Figure 1:**
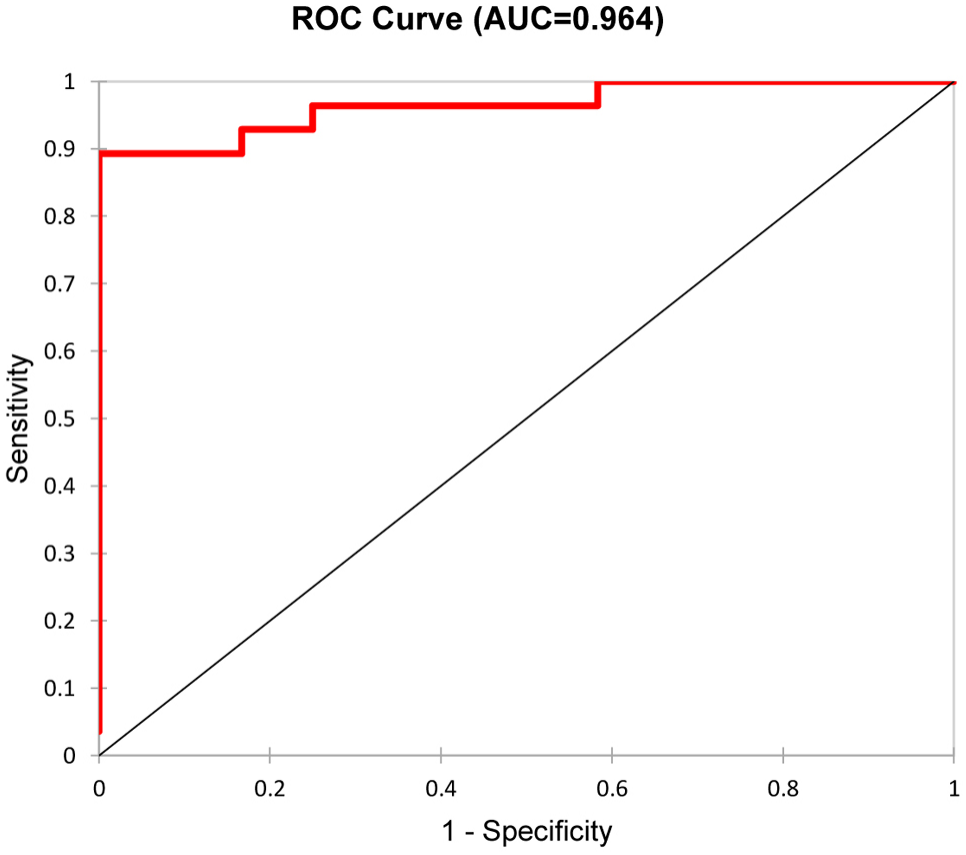
Discrimination of Focal class from the other classes with five selected urine biomarkers/ROC curve.

Kidney biopsies were classified as focal, crescentic, sclerotic and mixed class. Urinary DKK-3, CD163, EGF, PRO-C6 and C3M significantly differed in patients with different biopsy classes. These 5 urinary biomarkers were able to discriminate the focal class from the other classes (crescentic, sclerotic and mixed) with 90% accuracy (ROC 0.964 - **Figure 1**), or 92.5% accuracy with serum creatinine included. The urinary biomarkers were also able to discriminate the focal class from the crescentic class with 95.2% accuracy (**Table 3**) and the focal class from the sclerotic class with 95.4% accuracy (**Table 4**). We also tried to use the urinary biomarkers to differ the crescentic and sclerotic class. In this case, the accuracy of discrimination was not as high as in the previous measurements but the sensitivity of discrimination of these two classes was 100% (**Table 5**).

**Table 3:** AUC (from ROC), accuracy, sensitivity and specificity of the discrimination of Focal class from Crescentic class with selected urinary biomarkers.

Urinary biomarkers	AUC	Correct classification (accuracy), %	*Sensitivity, %	Specificity, %
**DKK-3**	0.833	71.4	80.0	68.8
**CD163**	0.843	76.2	83.3	73.3
**EGF**	0.944	85.7	80.0	90.9
**PRO-C6**	0.796	80.9	100.0	75.0
**C3M**	0.917	76.2	66.7	88.9
**5 urinary biomarkers**	1.000	95.2	100.0	92.3
**5 urinary biomarkers + serum creatinine**	1.000	100.0	100.0	100.0

*sensitivity to detect Focal against Crescentic.

**Table 4:** AUC (from ROC), accuracy, sensitivity and specificity of the discrimination of Focal class from Sclerotic class with selected urinary biomarkers.

Urinary biomarkers	AUC	Correct classification (accuracy), %	*Sensitivity, %	Specificity, %
**DKK-3**	0.842	68.2	80.0	64.7
**CD163**	0.492	54.5	50.0	55.0
**EGF**	0.950	86.4	81.8	90.9
**PRO-C6**	0.917	90.9	100.0	85.7
**C3M**	0.867	68.2	60.0	85.7
**5 urinary biomarkers**	1.000	95.4	100.0	92.3
**5 urinary biomarkers + serum creatinine**	1.000	100.0	100.0	100.0

*sensitivity to detect Focal against Sclerotic.

**Table 5:** AUC (from ROC), accuracy, sensitivity and specificity of the discrimination of Crescentic class from Sclerotic class with selected urinary biomarkers.

Urinary biomarkers	AUC	Correct classification (accuracy), %	*Sensitivity, %	Specificity, %
**DKK-3**	0.524	50.0	66.7	46.2
**CD163**	0.822	57.9	100.0	52.9
**EGF**	0.625	47.1	50.0	33.3
**PRO-C6**	0.544	47.4	50.0	47.1
**C3M**	0.611	57.9	66.7	53.8
**5 urinary biomarkers**	0.767	63.2	100.0	56.3
**5 urinary biomarkers + serum creatinine**	0.867	73.7	100.0	64.3

*sensitivity to detect Crescentic against Sclerotic.

The medians and I. and III. quartile of biomarker levels in particular biopsy classes are shown in **Table 6**.

**Table 6:** Biomarker concentration in serum and urine in the different bioptic classes (total N=40).

Biomarkers	Crescentic (N=9)	Focal (N=12)	Mixed (N=9)	Sclerotic (N=10)	Summary p-values
Serum creatinine* (µmol/l)	240(166;700)	104(84;118)	123(104;153)	221(191;450)	**<0.0001**
**Urinary DKK-3 (ng/mmol)**	1066 (515;1467)	169 (100;321)	557(318;1241)	1326(1056;2639)	**0.006**
**Urinary CD163 (ng/mmol)**	3753 (2197;4374)	568 (278;1261)	1133 (351;2253)	1010 (448;2124)	**0.021**
**Urinary EGF (ng/mmol)**	48 (30;93)	450 (334;950)	164 (91;320)	78 (51;106)	**<0.0001**
**Urinary PRO-C6 (ng/mg)**	25 (6;67)	7 (3;9)	16 (6;41)	55 (45;151)	**0.029**
**Urinary C3M (ng/mg)**	21 (11;29)	67 (45;113)	38 (21;48)	29 (17;43)	**0.002**

*HD substituted by the value 800.

Data are presented as median and I. and III. quartile. The bold p-values are significant.

The urinary DKK-3, EGF, PRO-C6 levels were also significantly related to the extent of renal fibrosis. While the urinary DKK-3 and PRO-C6 levels were highest in patients with more than 25% renal fibrosis (ci2+ci3, p-value for the comparison of ci0+ci1 vs. ci2+ci3 was 0.05 for urinary DKK3 and 0.01 for urinary PRO-C6), urinary EGF and C3M levels were highest in patients with less than 25% renal fibrosis (ci0+ci1) according to the Banff schema (p-value for the comparison of ci0+ci1 vs. ci2+ci3 was 0.025 for urinary EGF. However, p-value for urinary C3M was 0.1).

**Figure 2:**
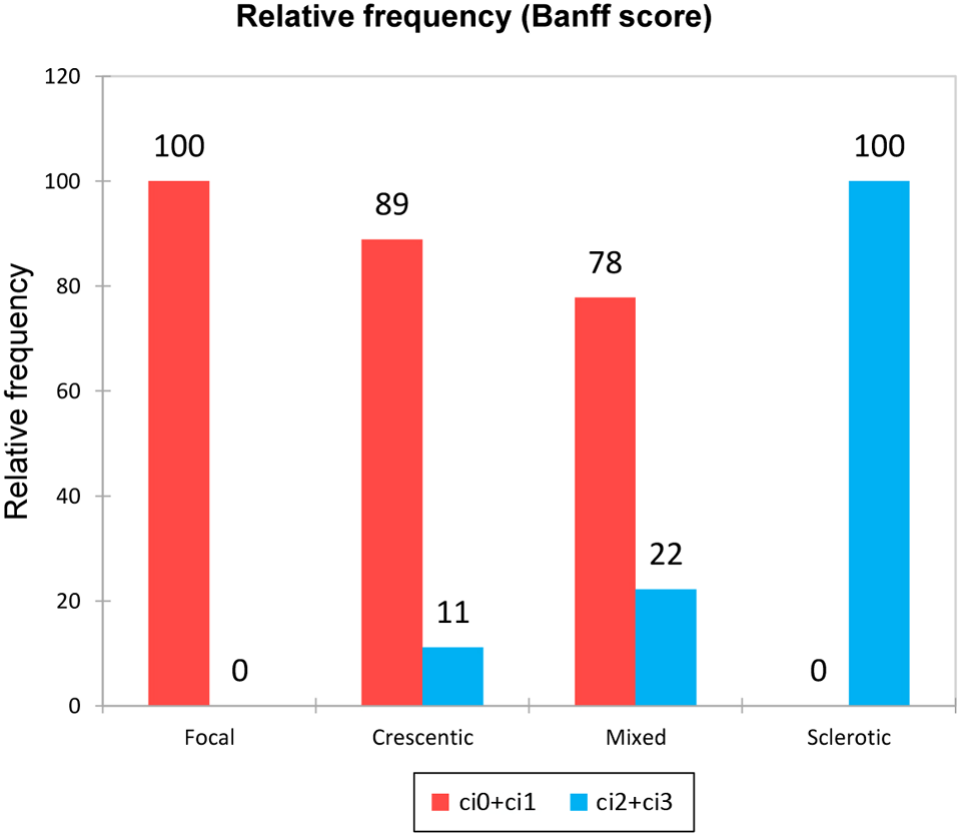
Extent of interstitial fibrosis in the different biopsy classes. According to Banff score: 1 – ci0+ci1; 2 – ci2+ci3.

Simultaneously, we were able to describe the association between the renal biopsy class and extent of renal fibrosis at the time of diagnosis. Unsurprisingly, while most patients with the focal and crescentic class had an interstitial fibrosis percentage less than 25% (Banff ci0+ci1), the sclerotic class displayed interstitial fibrosis of more than 25% (in the majority even more than 50% - Banff ci3) (**Figure 2**).

**Figure 3:**
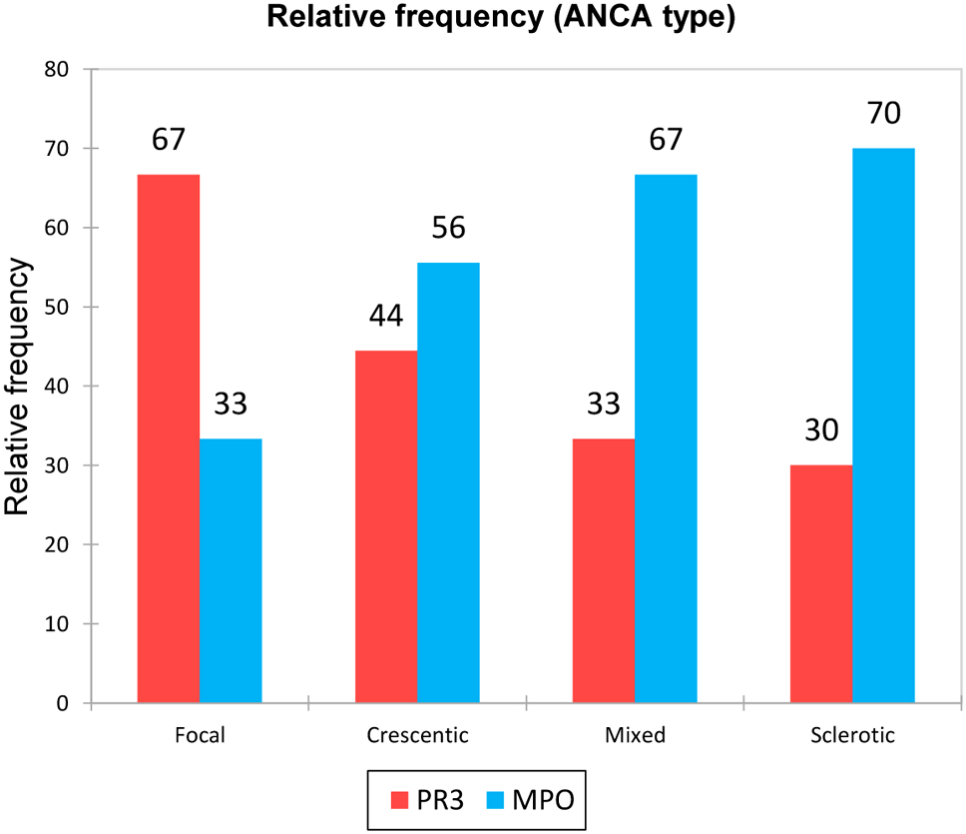
Relative frequency of ANCA type for the different biopsy classes.

We were interested in the representation of ANCA type for the different biopsy classes. ANCA MPO prevailed in the examined cohort and appeared mainly in patients with the sclerotic and mixed biopsy class. On the contrary, ANCA PR3 were represented mostly in the focal biopsy class (**Figure 3**). We also compared the biomarker results between the PR-3 and MPO-ANCA positive patients and did not find any statistically significant differences (p-values for all urinary biomarkers – DKK-3, CD163, EGF, PRO-C6, C3M were > 0.05).

### 3.4 Prognosis of final renal function

**Figure 4:**
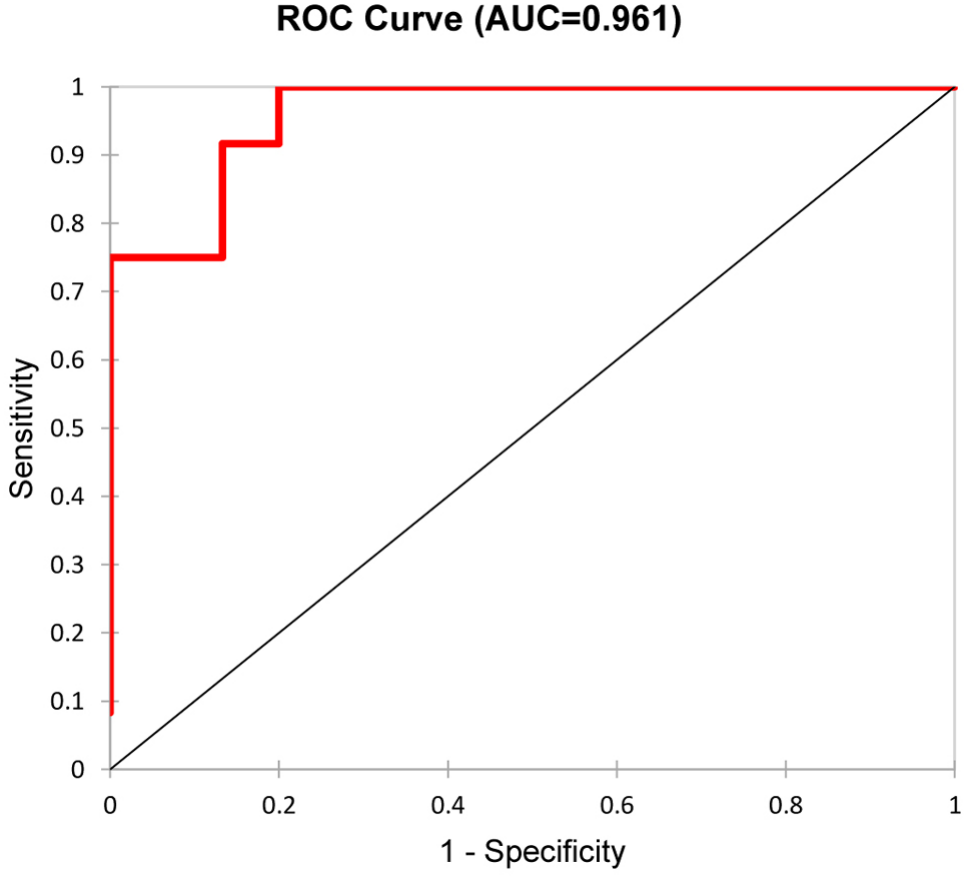
Differentiation of the two groups (favourable and unfavourable kidney outcome) by a combination of the five urinary biomarkers (DKK-3, CD163, EGF, PRO-C6, C3M).

The median time of follow-up was 38 months (range 12-71 months). The median of final serum creatinine was 156 (range 69-800) µmol/l in the whole cohort and 8 patients (18%) reached end-stage renal disease. Five urinary biomarkers (DKK-3, CD163, EGF, PRO-C6, C3M) collected at the time of diagnosis AAV were able to differentiate between patients with better or worse final renal function (favourable and unfavourable prognosis) at the end of follow-up with 88.9% accuracy (ROC 0.961, **Figure 4**), or 92.6% accuracy with serum creatinine included (**Table 7**). The levels of urinary DKK-3 and PRO-C6 collected at the time of renal biopsy were significantly higher in patients with worse final renal function at the end of follow-up. On the contrary, urinary EGF and C3M levels at entry were significantly higher in patients with better final renal function at follow-up. However, there was no significant association between urinary CD163 at entry and renal function at the end of follow-up (**Table 8**).

**Table 7:** AUC (from ROC), accuracy, sensitivity and specificity of the discrimination of the two groups (favourable and unfavourable kidney outcome) with 6 analysed biomarkers.

Biomarkers	AUC	Correct classification (accuracy), %	Sensitivity, %	Specificity, %
**Urinary DKK-3**	0.717	63.0	75.0	60.9
**Urinary CD163**	0.544	55.6	50.0	56.0
**Urinary EGF**	0.906	88.9	84.6	92.9
**Urinary PRO-C6**	0.883	74.1	87.5	70.0
**Urinary C3M**	0.744	70.4	83.3	66.7
**Serum creatinine at diagnosis**	0.861	66.7	66.7	66.7
**5 urinary biomarkers**	0.961	88.9	100.0	83.3
**5 urinary biomarkers + serum creatinine at diagnosis**	0.989	92.6	100.0	88.2

**Table 8:** Biomarker concentrations in urine at baseline in patients with favourable prognosis (serum creatinine at follow-up<156 µmol/l) and unfavourable prognosis (serum creatinine at follow-up>156 µmol/l, HD and Tx).

	Urinary DKK-3 (ng/mmol)	Urinary CD 163 (ng/mmol)	Urinary EGF (ng/mmol)	Urinary PRO-C6 (ng/mg)	Urinary C3M (ng/mg)
**Favourable prognosis**	277 (127;695)	1039 (371;2015)	333 (214;592)	7 (3;19)	54 (35;86)
**Unfavourable prognosis**	1108 (593;1701)	1690 (742;2876)	78 (33;106)	45 (13;86)	22 (12;39)
**p-values**	**0.045**	0.518	**0.0001**	**0.011**	**0.0001**

Data are presented as median and I. and III. quartile. N=38. The bold p-values are significant.

## 4 Discussion

In this study we examined a cohort of patients with AAV and a different extent of histological changes in their kidney biopsy in order to explore the potential association of selected biomarkers of fibrosis, inflammation and kidney tissue injury with biopsy findings and kidney function at the end of follow-up. We confirmed the diagnostic and prognostic value of selected urinary biomarkers in patients with AAV. Taken together, a panel consisting of 5 urinary biomarkers was able to clearly differentiate patients with renal AAV from healthy controls and together with routinely available serum creatinine was able to distinguish the biopsy classes with 92.5% accuracy.

Collagen type VI is a crucial ECM molecule for the control of tissue organization. It is present at the interface of the glomerular basement membrane and interstitial matrix and its levels have been reported elevated in glomeruli of patients with glomerular diseases (22). In previous studies, endotrophin, a fragment released during deposition of collagen VI and measured by the PRO-C6 assays, has shown profibrotic potential (23, 24). There is now an increasing interest in the role of PRO-C6 as a prognostic biomarker in different CKD settings (14, 16, 17, 25). In this study that complements and further expands results of our previous study in the same patient cohort (18), we observed increased urinary PRO-C6 levels in patients with AAV as compared with the healthy cohort. Furthermore, we proved the association between the high urinary PRO-C6 levels and more advanced renal biopsy findings (sclerotic class, more extensive interstitial fibrosis).

Similar to collagen type VI, collagen type III is an important ECM component in human body, and its imbalanced turnover is associated with CKD progression and advanced histological changes (15). We measured a collagen type III degradation biomarker (C3M) in serum and urine. In our cohort, especially urinary C3M levels were significantly higher in patients with less extensive histological changes in kidneys.

The levels of DKK-3, a stress-induced tubular epithelia-derived profibrotic glycoprotein, are significantly elevated in association with a higher degree of tubulointerstitial fibrosis in different glomerular and tubulointerstitial diseases (6) and they were related to progressive chronic kidney disease (7). Very similarly to PRO-C6, we observed that the urinary levels of DKK-3 increased with worse biopsy findings (e.g. in sclerotic class or extent of renal fibrosis more than 25%) in AAV.

Urinary soluble CD163 is a macrophage marker strongly expressed in glomerular crescents which was associated very tightly with active renal vasculitis (9, 10). In our cohort, urinary soluble CD163 levels were highest in patients with crescentic biopsy class, which indicates the most active renal vasculitis. In a previous study, patients with active ANCA GN had elevated urinary CD163 levels and the highest levels were observed in patients with crescentic biopsy class (10). Urinary soluble CD163 was also elevated in some patients with nonvasculitic forms of glomerulonephritis and was significantly elevated in active lupus nephritis (26).

The kidney is one of the major sites of EGF production. EGF is a very important marker for several biological functions, such as modulation of cell growth, renal repair following injury, regulation of cellular metabolism and glomerular haemodynamics (11). It is known that urinary EGF levels decrease in more severe renal disease. Low urinary EGF levels were associated with higher risk of progression to composite outcome in patients with AAV (27). Here we confirmed previous results, as we observed that patients with crescentic and sclerotic biopsy class had decreased urinary EGF levels.

We have used only urinary biomarkers (DKK-3, CD163, EGF, PRO-C6, C3M), as a fully non-invasive method, to distinguish respective biopsy classes. Although, the accuracy of discrimination with only urinary biomarkers was not as high as with serum and urinary biomarkers (data not shown), still the accuracy and sensitivity were high enough (80% and more). While the discrimination between the focal and the sclerotic class, and between the focal and the crescentic class reached 100% sensitivity and more than 90% specificity, distinguishing the crescentic from the sclerotic class proved to be less specific. We think that this might be caused by a high serum creatinine levels in both classes and found CD163 to be the most useful biomarker for this discrimination. In order to achieve higher accuracy and specificity, one would perhaps need to find yet another biomarker in the future. Furthermore, only the urinary DKK-3, EGF and PRO-C6 levels were significantly related to the extent of renal fibrosis.

As regards to ANCA levels in various biopsy classes, we observed higher ANCA PR3 levels in the focal class and higher ANCA MPO levels in the other biopsy classes, mainly in the sclerotic class. These findings could be explained by different manifestation of AAV with ANCA MPO and PR3. While ANCA MPO AAV is mostly connected with more gradual renal impairment and AAV is usually diagnosed later with more advanced histological changes, ANCA PR3 AAV is often diagnosed earlier due to extrarenal manifestation when renal impairment is not so advanced yet. We did not find any statistically significant differences in comparison between the biomarker levels and the PR-3 and MPO-ANCA positive patients.

We have also investigated the association of biomarker levels at the time of diagnosis and renal outcome at the end of follow-up. The 5 selected urinary biomarkers (DKK-3, CD163, EGF, PRO-C6 and C3M) significantly differed favourable and unfavourable group of patients. Urinary DKK-3 and PRO-C6 levels at the time of diagnosis were higher in patients with the worse renal function at the end of follow-up. On the contrary, urinary EGF and C3M levels were higher in patients with better renal function at the end of follow-up. Our findings support various studies in different renal diseases in which similar results had been observed (6, 8, 12, 14, 15, 28).

The limitations of this study include the inclusion of a relatively low number of patients in a single-center cohort, and a possible selection bias in the recruitment of the patients, as some of the patients were not included due to the lack of renal biopsy because of life-threatening disease symptoms, requiring acute hemodialysis or plasma exchange. Due to the design of the study and low number of patients, we were not able to confirm the results in a validation cohort, but plan to do that in the future. Moreover, we do not have any data on longitudinal biomarkers measurements that would elucidate the exact relationship of the markers to vasculitis activity, impact of treatment and kidney fibrosis ongoing development.

The strength of this study is that we have used biomarkers that can be easily assessed in biological fluids. Moreover, the urinary biomarkers identified in this study could be used as a non-invasive tool for monitoring histological and functional changes of kidney.One day, with further investigation and systematic assessment, the urinary biomarkers potentially can replace the renal biopsy, especially, they might be preferentially used where the biopsy was considered risky or could not be done due to aggressive disease course, or used for activity/chronicity assessment in cases with a suboptimal size of the biopsy samples, etc.

In conclusion, this small study demonstrated that non-invasive biomarkers of fibrosis, inflammation and kidney tissue injury were associated with severity of renal involvement and prognosis of kidney disease progression in patients with AAV.

## Data availability statement

The raw data supporting the conclusions of this article will be made available by the authors, without undue reservation.

## Ethics statement

The studies involving human participants were reviewed and approved by Ethics committee of General University Hospital in Prague. The patients/participants provided their written informed consent to participate in this study.

## Author contributions

ZH, VT, FG, NS and VS designed the study. MS performed the statistical analysis, made the figures and tables. VS drafted the paper. LB, EH, MK provided renal biopsy data. FG, NS, MA, HK provided laboratory data. VS, DF, RR, VB, ZH provided clinical data. ZH, VT, FG provided supervision. All authors contributed to the article and approved the submitted version.

## References

[1] Weidner S Geuss S Hafezi-Rachti S Wonka A Rupprecht HD. ANCA-associated vasculitis with renal involvement: An outcome analysis. Nephrol Dial Transplant. (2004) 19(6):1403–11. doi: 10.1093/ndt/gfh161 15069175

[2] Moiseev SV Novikov PI. Classification, diagnosis and treatment of ANCA-associated vasculitis. World J Rheumatol (2015) 5(1):36–44. doi: 10.5499/wjr.v5.i1.36

[3] Slot MC Tervaert JW Franssen CF Stegeman CA. Renal survival and prognostic factors in patients with PR3-ANCA associated vasculitis with renal involvement. Kidney Int (2003) 63(2):670–7. doi: 10.1046/j.1523-1755.2003.00769.x 12631133

[4] Moiseev S Novikov P Jayne D Mukhin N. End-stage renal disease in ANCA-associated vasculitis. Nephrol Dial Transplant. (2017) 32(2):248–53. doi: 10.1093/ndt/gfw046 28186571

[5] Berden AE Ferrario F Hagen EC Jayne DR Jennette JC Joh K. Histopathologic classification of ANCA-associated glomerulonephritis. J Am Soc Nephrol: JASN (2010) 21(10):1628–36. doi: 10.1681/ASN.2010050477 20616173

[6] Zewinger S Rauen T Rudnicki M Federico G Wagner M Triem S. Dickkopf-3 (DKK3) in urine identifies patients with short-term risk of eGFR loss. J Am Soc Nephrol. (2018) 29(11):2722–33. doi: 10.1681/ASN.2018040405 PMC6218861 30279273

[7] Federico G Meister M Mathow D Heine GH Moldenhauer G Popovic ZV. Tubular dickkopf-3 promotes the development of renal atrophy and fibrosis. JCI Insight (2016) 1(1). doi: 10.1172/jci.insight.84916 PMC5033928 27699213

[8] Fang X Hu J Chen Y Shen W Ke B. Dickkopf-3: Current knowledge in kidney diseases. Front Physiol (2020) 11:533344. doi: 10.3389/fphys.2020.533344 33391006 PMC7772396

[9] O’Reilly VP Wong L Kennedy C Elliot LA O'Meachair S Coughlan AM. Urinary soluble CD163 in active renal vasculitis. JASN (2016) 27(9):2906–16. doi: 10.1681/ASN.2015050511 PMC5004645 26940094

[10] Aendekerk JP Timmermans SAMEG Busch MH Potjewijd J Heeringa P Damoiseaux JGMC. Urinary soluble CD163 and disease activity in biopsy-proven ANCA-associated glomerulonephritis. Clin J Am Soc Nephrol. (2020) 15(12):1740–8. doi: 10.2215/CJN.07210520 PMC7769013 33203735

[11] Gesualdo L Di Paolo S Calabró A Milani S Maiorano E Ranieri E. Expression of epidermal growth factor and its receptor in normal and diseased human kidney: An immunohistochemical and *in situ* hybridization study. Kidney Int (1996) 49(3):656–65. doi: 10.1038/ki.1996.94 8648906

[12] Isaka Y. Epidermal growth factor as a prognostic biomarker in chronic kidney diseases. Ann Transl Med (2016) 4(Suppl 1):S62. doi: 10.21037/atm.2016.10.64 27868030 PMC5104645

[13] Bulanov N Chebotareva NV Novikov PI Moiseev SV. Role of tubulointerstitial injury in ANCA-associated vasculitis is underestimated. Ann Rheum Dis (2019) 78(10):e111. doi: 10.1136/annrheumdis-2018-214095 30061162

[14] Rasmussen DGK Fenton A Jesky M Ferro C Boor P Tepel M. Urinary endotrophin predicts disease progression in patients with chronic kidney disease. Sci Rep (2017) 7(1):17328. doi: 10.1038/s41598-017-17470-3 29229941 PMC5725589

[15] Genovese F Rasmussen DGK Karsdal MA Jesky M Ferro C Fenton A. Imbalanced turnover of collagen type III is associated with disease progression and mortality in high-risk chronic kidney disease patients. Clin Kidney J (2020) 14(2):593–601. doi: 10.1093/ckj/sfz174 33623684 PMC7886548

[16] Rasmussen DGK Hansen TW von Scholten BJ Nielsen SH Reinhard H Parving HH. Higher collagen VI formation is associated with all-cause mortality in patients with type 2 diabetes and microalbuminuria. Diabetes Care (2018) 41(7):1493–500. doi: 10.2337/dc17-2392 29643059

[17] Fenton A Jesky MD Ferro CJ Sørensen J Karsdal MA Cockwell P. Serum endotrophin, a type VI collagen cleavage product, is associated with increased mortality in chronic kidney disease. PloS One (2017) 12(4):e0175200. doi: 10.1371/journal.pone.0175200 28403201 PMC5389629

[18] Sparding N Genovese F Rasmussen DGK Karsdal MA Neprasova M Maixnerova D. Endotrophin, a collagen type VI-derived matrikine, reflects the degree of renal fibrosis in patients with IgA nephropathy and in patients with ANCA-associated vasculitis. Nephrol Dial Transplant. (2022) 37(6):1099–108. doi: 10.1093/ndt/gfab163 PMC9130028 33914059

[19] Genovese F Akhgar A Lim SS Farris AB Battle M Cobb J. Collagen type III and VI remodeling biomarkers are associated with kidney fibrosis in lupus nephritis. Kidney360 (2021) 2(9):1473–81. doi: 10.34067/KID.0001132021 PMC8786137 35373114

[20] Loupy A Haas M Solez K Racusen L Glotz D Seron D. The banff 2015 kidney meeting report: Current challenges in rejection classification and prospects for adopting molecular pathology. Am J Transplant. (2017) 17(1):28–41. doi: 10.1111/ajt.14107 27862883 PMC5363228

[21] Vandeginste BGM Massart DL Buydens LMC De Jong S Lewi PJ Smeyers-Verbeke J. Handbook of chemometrics and qualimetrics, part b. 1st ed. Elsevier (1998).

[22] Genovese F Rasmussen D Nielsen SH Haas J Voelker J Karsdal MA. A novel urinary biomarker of type VI collagen formation and endotrophin is associated with loss of kidney function in patients with diabetic nephropathy. Nephrol Dialysis Transplant (2017) 32(suppl_3):iii8. doi: 10.1093/ndt/gfx102

[23] Sun K Park J Gupta OT Holland WL Auerbach P Zhang N. Endotrophin triggers adipose tissue fibrosis and metabolic dysfunction. Nat Commun (2014) 5:3485. doi: 10.1038/ncomms4485 24647224 PMC4076823

[24] Lee C Kim M Lee JH Oh J Shin HH Lee SM. COL6A3-derived endotrophin links reciprocal interactions among hepatic cells in the pathology of chronic liver disease. J Pathol (2019) 247(1):99–109. doi: 10.1002/path.5172 30246318

[25] Tepel M Alkaff FF Kremer D Bakker SJL Thaunat O Nagarajah S. Pretransplant endotrophin predicts delayed graft function after kidney transplantation. Sci Rep (2022) 12(1):4079. doi: 10.1038/s41598-022-07645-y 35260630 PMC8904626

[26] Zhang T Li H Vanarsa K Gidley G Mok CC Petri M. Association of urine sCD163 with proliferative lupus nephritis, fibrinoid necrosis, cellular crescents and intrarenal M2 macrophages. Front Immunol (2020) 11:671. doi: 10.3389/fimmu.2020.00671 32351512 PMC7174755

[27] Wu L Li XQ Goyal T Eddy S Kretzler M Ju WJ. Urinary epidermal growth factor predicts renal prognosis in antineutrophil cytoplasmic antibody-associated vasculitis. Ann Rheum Dis (2018) 77(9):1339–44. doi: 10.1136/annrheumdis-2017-212578 29724728

[28] Rasmussen DGK Boesby L Nielsen SH Tepel M Birot S Karsdal MA. Collagen turnover profiles in chronic kidney disease. Sci Rep (2019) 9(1):16062. doi: 10.1038/s41598-019-51905-3 31690732 PMC6831687

